# Delay in admission for elective coronary-artery bypass grafting is associated with increased in-hospital mortality

**DOI:** 10.1186/1472-6963-8-185

**Published:** 2008-09-19

**Authors:** Boris G Sobolev, Guy Fradet, Robert Hayden, Lisa Kuramoto, Adrian R Levy, Mark J FitzGerald

**Affiliations:** 1 Department of Health Care and Epidemiology, The University of British Columbia, Vancouver, BC, Canada; 2 Department of Surgery, The University of British Columbia, Vancouver, BC, Canada; 3 Department of Surgery, Royal Columbian Hospital, New Westminster, BC, Canada; 4 Centre for Clinical Epidemiology and Evaluation, Vancouver Coastal Health Research Institute, Vancouver, BC, Canada

## Abstract

**Background:**

Many health care systems now use priority wait lists for scheduling elective coronary artery bypass grafting (CABG) surgery, but there have not yet been any direct estimates of reductions in in-hospital mortality rate afforded by ensuring that the operation is performed within recommended time periods.

**Methods:**

We used a population-based registry to identify patients with established coronary artery disease who underwent isolated CABG in British Columbia, Canada. We studied whether postoperative survival during hospital admission for CABG differed significantly among patients who waited for surgery longer than the recommended time, 6 weeks for patients needing semi-urgent surgery and 12 weeks for those needing non-urgent surgery.

**Results:**

Among 7316 patients who underwent CABG, 97 died during the same hospital admission, for a province-wide death rate at discharge of 1.3%. The observed proportion of patients who died during the same admission was 1.0% (27 deaths among 2675 patients) for patients treated within the recommended time and 1.5% (70 among 4641) for whom CABG was delayed. After adjustment for age, sex, anatomy, comorbidity, calendar period, hospital, and mode of admission, patients with early CABG were only 2/3 as likely as those for whom CABG was delayed to experience in-hospital death (odds ratio 0.61; 95% confidence interval [CI] 0.39 to 0.96). There was a linear trend of 5% increase in the odds of in-hospital death for every additional month of delay before surgery, adjusted OR = 1.05 (95% CI 1.00 to 1.11).

**Conclusion:**

We found a significant survival benefit from performing surgical revascularization within the time deemed acceptable to consultant surgeons for patients requiring the treatment on a semi-urgent or non-urgent basis.

## Background

In health care systems that use surgical wait lists to contain costs, there is a major concern that delays in necessary procedures may lead to deterioration in the patient's condition, poor clinical outcome, and increased risk of death [1-3]. In Canada and other countries, establishing a recommended time that patients can safely wait for a particular operation is generally perceived as a suitable method for preventing the adverse outcomes associated with treatment delay [4,5]. For example, priority wait lists are commonly used for queuing patients with coronary artery disease who are to undergo coronary artery bypass grafting (CABG) [6,7]. In theory, queuing procedures should ensure access to care according to the severity of the underlying condition [8]. However, admission for elective (i.e., non-emergency but necessary) surgery within the recommended time can be easily affected if surgical services experience an uneven influx of more urgent cases [9]. We previously performed an empirical analysis of a population-based registry and found that the length of the queue at the time of a patient's registration also affected the time to surgery [10], the probability of surgery being delayed [11], and the probability of death before planned surgery [12].

The proportion of patients who die after surgery but during the same hospital admission is an important indicator of the quality of surgical care [13,14]. It may also reflect the impact of a delay in surgery [15]. Surprisingly, few studies have correlated the in-hospital mortality rate with timing of surgery after registration for elective CABG [16], yet this relationship is important in deciding how much capacity is required to avoid unacceptable delays that put patients at an increased risk of postoperative death. In addition, it has been argued that cardiologists have a duty to inform their patients of the likely extent of treatment delay and associated risks when referring a patient for consultation with cardiac surgeon [17]. To date, however, there are no direct estimates of reduction in postoperative in-hospital mortality afforded by performing CABG within the time deemed acceptable to consultant surgeons.

In this population-based study, we examined whether postoperative in-hospital mortality during hospital admission for CABG differed significantly among patients who waited for the operation longer than recommended by the consensus of cardiac surgeons in the Canadian province of British Columbia. The study cohort included only patients who had undergone a single operation, first-time isolated CABG, after registration on a wait list; and the time between registration and surgery was a study variable. The protocol for this study was approved by the University of British Columbia Ethics Board.

## Methods

### Data sources

The data were obtained from the British Columbia Cardiac Registry (BCCR). This database, created in 1991, prospectively collects information about dates of registration, procedure, or withdrawal and about disease severity and other risk factors for all patients registered to undergo surgical coronary revascularization in any of the four tertiary-care hospitals that provide cardiac care to adult residents of British Columbia [10]. To identify hospital admission and discharge dates, coexisting medical conditions, and in-hospital deaths, we used each patient's provincial health number to link deterministically BCCR records to the British Columbia Linked Health Database Hospital Separations File [18]. To identify coexisting medical conditions in the study cohort we retrieved diagnoses reported in discharge abstracts within 1 year before registration for CABG [19].

### Patients

The inception cohort consisted of all adult British Columbia residents with established coronary artery disease and for whom a request from a cardiac surgeon to book an operating room for isolated CABG in one of the participating hospitals was recorded between January 1, 1991, and December 31, 2000. The patients included in this study were those who underwent treatment on a semi-urgent or non-urgent basis and who had not previously undergone CABG (see Table 1 for criteria). A total of 54 records were excluded, either because the registration and procedure dates were identical (*n *= 50) or because there was no surgical report (*n *= 4). We excluded an additional 115 records for which registration and admission dates suggested that the procedure had been performed immediately. In these 169 excluded records, the prevalent age group was 60 to 69 years old (42%); there were more men (81%), semi-urgent patients (91%), those who had multi-vessel disease (83%) and co-existing conditions (75%). Among the excluded, three in-hospital deaths were recorded. The final study cohort consisted of 7316 patients who had undergone first-time isolated (did not include a valve replacement procedure) CABG.

**Table 1:** Definition of study groups

Group	Target Time for Surgery	Anginal Symptoms, Coronary Anatomy, and Left Ventricular Function
Semi-urgent	6 weeks	Patients with either persistent unstable angina or stable angina and extensive coronary artery disease (left-main stenosis more than 50%, triple-vessel disease, or double-vessel disease with significant proximal left anterior descending stenosis and impaired left ventricular function)
Non-urgent	12 weeks	Stable symptomatic patients with limited coronary artery disease (double-vessel disease with no lesion in the proximal left anterior descending artery and normal left ventricular function or single-vessel disease with significant stenosis of the proximal left anterior descending artery)

We did not include in the study cohort patients who were removed from the wait lists without surgery for various reasons: died while awaiting surgery (1.0%), became unfit to surgery (2.2%), declined surgery (2.4%), underwent surgery elsewhere (1.2%), or underwent other surgery (0.3%).

When accepting patients for CABG, the 18 cardiac surgeons used a common algorithm to set the urgency for booking the operating room, taking account of the severity of patient's condition and considerations of expected benefit, such that the operation could be performed within a clinically appropriate time (shown in Table 1), as previously described [20]. In this analysis, a patient's need for treatment was classified as semi-urgent if the suggested time to surgery was within 6 weeks after the treatment decision had been made or "non-urgent" if that time was within 12 weeks.

### Primary outcome and study variables

The primary outcome was postoperative in-hospital death, i.e., any death that occurred after the operation but during the same hospital admission [15]. Postoperative discharge to another hospital was counted as discharge alive. The time to treatment was computed as the number of calendar weeks from registration on a wait list to surgery. The date of the surgeon's request to book the operating room was used as the date of registration on the wait list. Patients who waited for CABG longer than the recommended time (6 or 12 weeks, depending on urgency) were classified as having undergone late surgery. Those who waited less than the recommended time were classified as having undergone early surgery.

Although the urgency of treatment was changed between time of registration and time of surgery for 19% patients, we used the urgency at registration for classifying patients because there was no record of the timing of these changes. We added the mode of admission in regression analysis to acknowledge that early surgery may be a result of unplanned emergency admission [21].

### Statistical analysis

We used proportions to characterize the probability that a postoperative death occurred during the hospital admission and chi-square testing to compare the proportions between patients who had early and late surgery. The effect of early surgery on the odds of postoperative in-hospital death was estimated by means of logistic regressions, which yielded the odds ratio (OR) as a measure of effect size. The timing of surgery was entered as an indicator variable, with a value of 1 denoting a wait time within the recommended period. The exponential of the regression coefficient for that variable gives the OR of in-hospital deaths for early relative to late surgery. In a separate analysis the impact of delaying the treatment was evaluated for every additional month of delay using a continuous variable for the number of months between registration on a wait list and surgery.

In the multivariate analysis, we controlled for differences in patients' characteristics and significant confounders (summarized in Table 2). Existing literature suggests that elderly patients are more likely to undergo revascularization as an urgent procedure [22]; that smaller coronary vessel diameters may account for the higher risk of adverse events in women [23]; that coexisting medical conditions may delay open heart surgery [6]; and that changes in practice and available funding may reduce the time to surgery [20]. In particular, we entered two indicator variables for three comorbidity categories. The reference category was no coexisting conditions and the two comparison categories were presenting with congestive heart failure, diabetes mellitus, chronic obstructive pulmonary disease, cancer, or rheumatoid arthritis [24] and presenting with other coexisting chronic conditions as defined elsewhere [25]. We adjusted for the hospital where the patient was treated, to address the potential influence on outcome of standards of anesthesia, surgery, and intensive care; adequacy of the facilities and staffing levels; attitude to training; and interpersonal relationships among staff [15]. We used the period of registration for surgery as a proxy of changes in practice and available funding.

**Table 2:** Characteristics of 7316 wait-listed patients who underwent isolated coronary artery bypass surgery in British Columbia 1991–2001.

Characteristic	Early surgery, N (%)	Late surgery, N (%)
Age group (yr)		
40–49	223 (8.3)	375 (8.1)
50–59	552 (20.6)	1075 (23.2)
60–69	1021 (38.2)	1772 (38.2)
70–79	830 (31.0)	1348 (29.0)
80 and over	49 (1.8)	71 (1.5)
		
Sex		
Female	482 (18.0)	791 (17.0)
Male	2193 (82.0)	3850 (83.0)
		
Urgency at registration		
Semi-urgent	2107 (78.8)	3685 (79.4)
Non-urgent	568 (21.2)	956 (20.6)
		
Comorbidity at registration		
Major conditions*	603 (22.5)	926 (20.0)
Other conditions†	785 (29.3)	1096 (23.6)
None	1287 (48.1)	2619 (56.4)
		
Affected anatomy		
Left main	403 (15.1)	505 (10.9)
Multi-vessel‡	2054 (76.8)	3809 (82.1)
Limited§	218 (8.1)	327 (7.0)
		
Period of registration		
1991–1992	633 (23.7)	710 (15.3)
1993–1994	687 (25.7)	851 (18.3)
1995–1996	354 (13.2)	1122 (24.2)
1997–1998	450 (16.8)	1058 (22.8)
1999–2000	551 (20.6)	900 (19.4)
		
Hospital		
1	662 (24.7)	831 (17.9)
2	1004 (37.5)	1507 (32.5)
3	390 (14.6)	1270 (27.4)
4	619 (23.1)	1033 (22.3)
		
Mode of admission		
Elective	2524 (94.4)	4526 (97.5)
Unplanned emergency	151 (5.6)	115 (2.5)

* Congestive heart failure, diabetes mellitus, chronic obstructive pulmonary disease, cancer, or rheumatoid arthritis.

†Peripheral vascular disease, cerebrovascular disease, dementia, peptic ulcer disease, hemiplegia, renal disease, or liver disease.

‡Three- or two-vessel disease with stenosis of the proximal left anterior descending artery.

§Two-vessel disease with no lesion in the proximal left anterior descending artery or single-vessel disease with stenosis of the proximal left anterior descending artery.

## Results

### Patients

Table 2 shows the distribution of elective patients who underwent isolated coronary artery bypass surgery according to age, sex, urgency at registration, coexisting conditions, coronary anatomy, calendar period, hospital, and mode of admission. Age distribution was similar in the early and late surgery groups, with the majority (68%) undergoing surgery between 60 and 79 years of age. The distribution of sex and urgency was also similar between the two groups, the majority being male (83%) and needing semi-urgent treatment (79%). Differences between these two groups in terms of coexisting medical conditions and coronary anatomy indicate that sicker patients were more likely to undergo the procedure without delay. For example, 56% of patients who underwent late surgery but only 48% of those who underwent early surgery had no coexisting conditions (*P *< 0.001). The proportion of patients undergoing surgery after the recommended time increased from 53% before 1995–1996 to 76% after 1995–1996 (*P *< 0.001). Unplanned emergency admissions were more common in the group of patients who underwent early surgery (5.6% and 2.5%, respectively, *P *< 0.001).

### In-hospital postoperative mortality rate

Among the 7316 patients who underwent CABG, 97 patients died postoperatively during the same hospital admission, for a province-wide death rate at discharge of 1.3%. There were 27 in-hospital deaths among the 2675 patients who underwent early surgery and 70 deaths among the 4641 patients who underwent late surgery (Table 3). The observed proportion of patients who died during the same admission was 1.0% (95% confidence interval [CI] 0.6% to 1.4%) for patients treated within the recommended time and 1.5% (95% CI 1.2% to 1.9%) for whom CABG was delayed.

**Table 3:** Association between postoperative in-hospital mortality and treatment delay as measured by odds ratios (OR) derived from logistic regression models

Factor	Death/Surgery	% (95% CI)	Unadjusted OR	Adjusted OR*
Timing of surgery				
Late	70/4641	1.5 (1.2–1.9)	1.00	1.00
Early	27/2675	1.0 (0.6–1.4)	0.67 (0.43–1.04)	0.61 (0.39–0.96)
				
Urgency at registration				
Semi-urgent	11/1524	0.7 (0.3–1.1)	1.00	1.00
Non-urgent	86/5792	1.5 (1.2–1.8)	2.07 (1.10–3.89)	1.63 (0.84–3.18)

*Adjusted for age, sex, anatomy, comorbidity, calendar period, hospital, and mode of admission

Table 3 shows the relationship between the timing of surgery and in-hospital mortality as measured by odds ratios. Without adjustment for other factors, patients with early CABG were only 2/3 as likely as those for whom CABG was delayed to experience in-hospital death, unadjusted OR = 0.67 (95% CI 0.43 to 1.04). After adjustment for age, sex, anatomy, comorbidity, calendar period, hospital, and mode of admission, the difference between the two groups was statistically significant, adjusted OR = 0.61 (95% CI 0.39 to 0.96).

Table 3 also shows the association between in-hospital mortality and urgency. The proportion of patients who died postoperatively was 1.5% (95% CI 1.2% to 1.8%) for patients needing semi-urgent treatment and 0.7% (95% CI 0.3% to 1.1%) for those needing non-urgent treatment. Without adjustment for other factors, patients needing semi-urgent surgery were twice as likely as those needing non-urgent surgery to experience postoperative death, OR = 2.07 (95% CI 1.10 to 3.89). After adjustment, the likelihood of death remained substantially higher, although not reaching statistical significance, OR = 1.63 (95% CI 0.84 to 3.18).

The impact of the timing of surgery was also evaluated for every additional month of delay. The regression analysis suggests a linear trend of a 5% increase in the odds of in-hospital death for every additional month of delay before surgery, adjusted OR = 1.05 (95% CI 1.00 to 1.11).

## Discussion and conclusion

Many health care systems now use priority wait lists for scheduling elective CABG, but there have not yet been any direct estimates of reductions in in-hospital mortality rate afforded by ensuring that the operation is performed within a clinically appropriate time. We conducted this study on a data set representing all British Columbia patients undergoing first-time isolated CABG after registration on wait lists on a semi-urgent and non-urgent basis over a 10-year period. Patients who underwent the operation without registration on a CABG wait list and patients who were removed from the wait lists without surgery were not included in this analysis.

The contribution of this paper is two-fold. First, we have reported direct estimates of a reduction in postoperative in-hospital mortality, which can be achieved by completing a first-time isolated CABG within the time deemed acceptable to consultant surgeons. Second, we found that among patients who underwent the operation within the recommended times postoperative in-hospital death was only 2/3 as likely as among those who had to wait longer.

The Hospital Insurance and Diagnostics Act (1957), the Medical Insurance Act (1967), and the Canada Health Act (1984) laid the groundwork for a publicly administered health care system in Canada that is universal, accessible, portable, and comprehensive. As others have pointed out, the principle of accessibility was not originally intended to address the timeliness of access to elective care [17]. Wait lists for elective surgery have therefore been commonly accepted in Canada on the premise that they ensure the most efficient use of hospital resources; the only alternative would be to have under utilization of capacity within the health care system [8]. The health system's operational considerations alone may be irrelevant, as the Supreme Court of Canada agreed in a recent ruling that long health-care wait times constitute deprivation of rights to personal inviolability guaranteed by the Charter of Rights and Freedoms [26]. The debate has therefore shifted toward the issue of how much capacity is required to maintain safe wait lists [17]. The Canadian federal government called for establishing target access times for open-heart surgery that would minimize the adverse events associated with treatment delay. Although there is a considerable amount of literature on risks of adverse events while waiting for CABG, only a few studies have reported on the effect of delays in admission on postoperative mortality, an important measure of performance. The analysis of postoperative deaths reported here provides further support for keeping the wait lists for elective CABG short. We found a significant survival benefit of performing early surgical revascularization for patients requiring the treatment on a semi-urgent or non-urgent basis.

Other investigators reported that they found no evidence for an increase in 30-day postoperative mortality rate among patients with prolonged waiting time before CABG [16]. Several differences between that study and the one reported here might have contributed to the discrepancy between their conclusions and ours. First, we studied a single operation, a first-time isolated CABG, whereas the other researchers reported on multiple procedures including valve replacement. Second, we studied postoperative death during admission for the index operation [15], whereas they reported on 30-day mortality. Third, we adjusted the effect size estimates for important confounders. Finally, we used the priority assigned at the time of acceptance for surgery, whereas they determined priority at the time of surgery. Other researchers reported no difference between patients undergoing early and late surgery in terms of postoperative mortality rates after multiple different cardiac procedures [3], whereas another group working with a smaller cohort found that longer waiting times were associated with more postoperative adverse events [27].

Although the data have been validated locally at each hospital, no external audit was performed to evaluate the quality of the registry records. Misclassification of the recorded urgency for treatment is a potential concern in this analysis, because surgeons may select patients from wait lists based on other considerations, such as best use of operating time or the availability of hospital resources. Another concern is that for some patients, the urgency was reclassified at the time of surgery, which probably reflects a deterioration in clinical status. However, there was no record of the timing of these changes between registration and surgery. Therefore, in regression analysis we adjusted for the mode of admission because early surgery may be a result of unplanned emergency admission. The data set did not have enough events to allow adjustment for the surgical operator, and outcomes might have been influenced by the individual surgeon's threshold for accepting a patient for non-urgent treatment [15]. Similarly, we were unable to adjust for a possible imbalance in urban versus rural referrals between the study groups. In spite of these limitations, our findings suggest a significant survival benefit from performing surgical revascularization within the time deemed acceptable to consultant surgeons for patients requiring the treatment on a semi-urgent or non-urgent basis.

## Competing interests

The authors declare that they have no competing interests.

## Authors' contributions

BS conceived the study concept and design, participated in analysis and interpretation, and drafted the manuscript. GF participated in data acquisition and critically revised the manuscript. RH participated in data acquisition. LK performed statistical analysis and drafted the manuscript. AL participated in data acquisition. MF critically revised the manuscript and has been involved in drafting the manuscript. All authors read and approved the final manuscript.

## Pre-publication history

The pre-publication history for this paper can be accessed here:


